# Supplementary material for: Geometrical Constraints on the Tangling of Bacterial Flagellar Filaments
Source: Sci Rep. 2020 May 21;10:8406. doi: 10.1038/s41598-020-64974-6 (PMC7242426; doi:10.1038/s41598-020-64974-6)
Supplement: Supplementary file 2 — Supplementary Information [file 41598_2020_64974_MOESM2_ESM.pdf]

Supplementary Material for "Geometrical Constraints on the Tangling of Bacterial Flagellar Filaments"

Maria Tătulea-Codrean* and Eric Lauga†
Department of Applied Mathematics and Theoretical Physics,
University of Cambridge, Cambridge CB3 0WA, United Kingdom
(Dated: April 9, 2020)

CONTENTS

I. Choice of focal plane 1

II. Calculation of cross-sectional shape 1

III. Effect of tapering and anchoring 5

IV. Sources of experimental data 6

V. Video descriptions 7
Video S1 7
Video S2 7
Video S3 7

References 7

I. CHOICE OF FOCAL PLANE

We are interested in visualising the point of contact between the helical tube and the vertical z-axis, and in order to do so we must look in the plane normal to $\mathbf{e}_2(\phi) = (-\sin\phi, \cos\phi, 0)$ going through the origin, see Fig. S1. If any point on the helical tube is in contact with the vertical z-axis, then it will necessarily have zero component along $\mathbf{e}_2$, as does every point on the z-axis. Indeed, points on the z-axis have zero component along any vector orthogonal to $\mathbf{e}_z$, but it is convenient to look in this plane because it is parallel to the helical axis, as $\mathbf{e}_3 \cdot \mathbf{e}_2 = 0$.

If one imagines keeping the azimuthal angle, ϕ, fixed and varying the incline angle, θ, in order to find the special value $\hat{\theta}(\phi, s)$ at which the helical tube is tangent to the z-axis at point s along the helix, then the cylindrical envelope containing the helix will be "sliced" by the z-axis along the plane normal to $\mathbf{e}_2(\phi) = (-\sin\phi, \cos\phi, 0)$ going through the origin. We must then consider the intersection of the helical tube with this plane in order to find the point where the filament is exactly tangent to the line, essentially solving for $\hat{\theta}(\phi, s)$. Then, by asking that the filament is tangent to the line again at $s - p$, we will determine $s_{\text{crit}}(\phi)$ and $\theta_{\text{crit}}(\phi) = \hat{\theta}(\phi, s_{\text{crit}}(\phi))$. These derivations are explained in the main manuscript.

II. CALCULATION OF CROSS-SECTIONAL SHAPE

We want to determine the cross-sectional shape of the helical filament as it intersects the focal plane. For our analytical model we only consider perfect helices with constant helical amplitude, r, while tapering is handled numerically. As in the main manuscript, we parameterise the helical centreline by s, such that

$$\mathbf{r}_c(s) = r\cos\left(\frac{2\pi s}{p}\right)\mathbf{e}_1 - r\sin\left(\frac{2\pi s}{p}\right)\mathbf{e}_2 + s\mathbf{e}_3 + \frac{d}{2}\mathbf{e}_x, \tag{S1}$$

---

* m.tatulea-codrean@damtp.cam.ac.uk
† e.lauga@damtp.cam.ac.uk

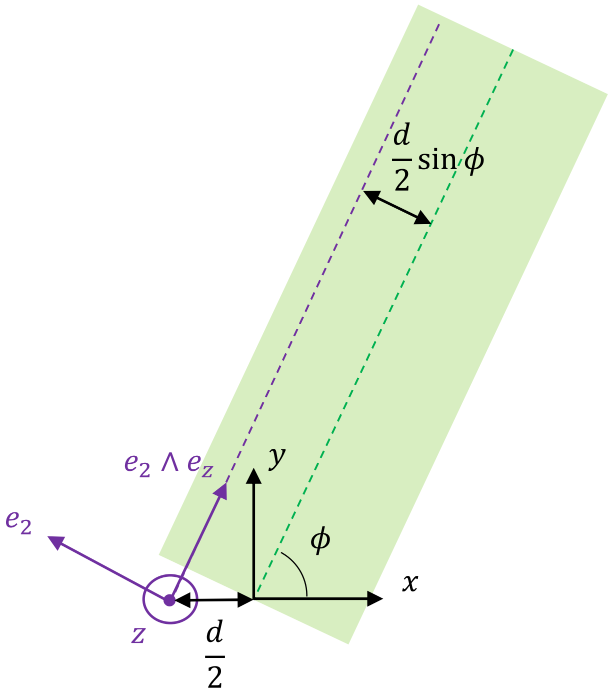

FIG. S1. Projection on the xy-plane. The projection of the cylindrical envelope containing the helix is shown in light green. The direction of the vector $\mathbf{e}'_x = (\cos\phi, \sin\phi, 0)$ is given by a dashed dark purple line. It is parallel to the projection on the xy-plane of the helical axis and lies at a distance $d\sin\phi/2$ away from it. The "focal plane" is normal to the vector $\mathbf{e}'_y = (-\sin\phi, \cos\phi, 0) = \mathbf{e}_2(\phi)$ and goes through the origin, denoted by a dark purple dot. The helix is anchored to the xy-plane at a distance $d/2$ away from the origin along the x-axis.

where we have added the displacement $d\mathbf{e}_x$ between the anchoring points of the two helices (corresponding to a displacement $d/2\mathbf{e}_x$ between one helix and the z-axis, which is also the axis of rotational symmetry). Without loss of generality, we present the calculations for a left-handed helix with $\sigma = -1$ and phase $\chi = 0$ (compare with Eq. (3)). The tangent vector to the centreline is

$$\hat{\mathbf{t}}(s) = -\sin\psi \sin\left(\frac{2\pi s}{p}\right)\mathbf{e}_1 - \sin\psi\cos\left(\frac{2\pi s}{p}\right)\mathbf{e}_2 + \cos\psi\mathbf{e}_3, \tag{S2}$$

where $\psi = \tan^{-1}(2\pi r/p)$ is the pitch angle of the helix. Similarly, we have the normal and the binormal vectors

$$\hat{\mathbf{n}}(s) = -\cos\left(\frac{2\pi s}{p}\right)\mathbf{e}_1 + \sin\left(\frac{2\pi s}{p}\right)\mathbf{e}_2, \tag{S3}$$

$$\hat{\mathbf{b}}(s) = -\cos\psi\sin\left(\frac{2\pi s}{p}\right)\mathbf{e}_1 - \cos\psi\cos\left(\frac{2\pi s}{p}\right)\mathbf{e}_2 - \sin\psi\mathbf{e}_3. \tag{S4}$$

Assuming that the filament has a circular cross-section of radius r_ϵ, the outside surface of the filament can be parameterised by two variables (s, ξ) in the following way

$$\mathbf{r}(s,\xi) = \mathbf{r}_c(s) + r_\epsilon\left(\cos\xi\hat{\mathbf{n}}(s) + \sin\xi\hat{\mathbf{b}}(s)\right). \tag{S5}$$

The intersection between the filament surface and the focal plane will be given by the points that satisfy

$$\mathbf{r}(s,\xi)\cdot\mathbf{e}_2 = 0, \tag{S6}$$

by construction of the focal plane. This can be rearranged into

$$(r - r_\epsilon \cos\xi)\sin\left(\frac{2\pi s}{p}\right) + r_\epsilon \sin\xi\cos\psi\cos\left(\frac{2\pi s}{p}\right) = -\frac{d}{2}\sin\phi. \tag{S7}$$

Using double angle formulae, we can find the solution to the above equation as a one-parameter family of points $s = s^*(\xi)$ that satisfy

$$s^*(\xi) = \frac{p}{2\pi}\left[\sin^{-1}\left(\frac{-d\sin\phi}{2\sqrt{(r - r_\epsilon\cos\xi)^2 + (r_\epsilon\sin\xi\cos\psi)^2}}\right) - \tan^{-1}\left(\frac{r_\epsilon\sin\xi\cos\psi}{r - r_\epsilon\cos\xi}\right)\right]. \tag{S8}$$

Inverse trigonometric functions are multivalued, and the solutions correspond to different intersections between the helix and the focal plane. In this case, the inverse tangent must always be evaluated in an interval with positive cosine, since $r - r_\epsilon\cos\xi > 0$ for all ξ. There are two possible values for the inverse sine which correspond to the helix intersecting the focal plane twice per helical turn – once on the left of the helical axis and once on the right, see Fig. 3(a). We continue our calculations with the branch of solutions where the inverse sine is evaluated in the interval $(\pi/2, 3\pi/2)$, because this describes the cross-sectional shape seen in Fig. 3(c). The other branch of solutions would give the intersections of the helix with the focal plane on the other side of the helical axis, but these are not shown in Fig. 3(c) because they are not important for the model.

With this in mind, we can Taylor expand Eq. (S8) for $r_\epsilon \ll r$ to give

$$s^*(\xi) = \frac{p}{2\pi}\left[\pi + \phi' + \frac{r_\epsilon}{r}\tan\phi'\cos\xi - \frac{r_\epsilon}{r}\cos\psi\sin\xi + \mathcal{O}\left(\left(\frac{r_\epsilon}{r}\right)^2\right)\right], \tag{S9}$$

where we have used the notation $\phi' = \sin^{-1}(d\sin\phi/2r)$ as in the main manuscript.

The outline of the filament in the focal plane is simply the locus of points $\mathbf{r}(s^*(\xi), \xi)$. We begin by computing this shape in terms of coordinates along the vectors $\{\mathbf{e}_1, \mathbf{e}_3\}$ which span the focal plane and are perpendicular and, respectively, parallel to the helical axis. Using Eqs. (S1)-(S5) and the orthonormality of the basis $\{\mathbf{e}_1, \mathbf{e}_2, \mathbf{e}_3\}$ we deduce that

$$\mathbf{r}(s^*(\xi), \xi)\cdot\mathbf{e}_1 = (r - r_\epsilon\cos\xi)\cos\left(\frac{2\pi s^*}{p}\right) - r_\epsilon\cos\psi\sin\xi\sin\left(\frac{2\pi s^*}{p}\right), \tag{S10}$$

$$\mathbf{r}(s^*(\xi), \xi)\cdot\mathbf{e}_3 = s^*(\xi) - r_\epsilon\sin\psi\sin\xi. \tag{S11}$$

Substituting the approximate expression for $s^*(\xi)$ from Eq. (S9), we get that

$$\mathbf{r}(s^*(\xi), \xi)\cdot\mathbf{e}_1 \approx -r\cos\phi' + \frac{r_\epsilon}{\cos\phi'}\cos\xi, \tag{S12}$$

$$\mathbf{r}(s^*(\xi), \xi)\cdot\mathbf{e}_3 \approx \frac{p(\phi' + \pi)}{2\pi} - \frac{r_\epsilon}{\sin\psi}\sin\xi + \frac{r_\epsilon\tan\phi'}{\tan\psi}\cos\xi. \tag{S13}$$

The first term in each equation represents the leading-order position of the centreline, while the other terms are an approximation to first order in r_ϵ/r of the cross-sectional shape of the filament in the focal plane. After some algebra, we can manipulate the first-order terms into the form

$$r_\epsilon\cos(\xi - \xi_0)\begin{pmatrix}\cos\alpha \\ -\sin\alpha\end{pmatrix} - \frac{r_\epsilon}{\sin\psi\cos\phi'}\sin(\xi - \xi_0)\begin{pmatrix}\sin\alpha \\ \cos\alpha\end{pmatrix}, \tag{S14}$$

where we have introduced the simplifying notation

$$\xi_0 = \cot^{-1}(\cot\phi'\cos\psi), \tag{S15}$$

$$\alpha = \tan^{-1}(\sin\phi'\tan\psi). \tag{S16}$$

By writing the first-order terms in this form, we can clearly see that the parameter ξ traces out an ellipse with principal axes parallel to the two column vectors appearing in Eq. (S14). Therefore, the right-hand side of Eqs. (S12)-(S13) is an exact parameterisation of an ellipse centred at $-r\cos\phi'\mathbf{e}_1 + p(\phi' + \pi)/2\pi\mathbf{e}_3$ with minor axis length, $2a$, and major axis length, $2b$, given by

$$a = r_\epsilon, \quad b = \frac{r_\epsilon}{\sin\psi\cos\phi'}. \tag{S17}$$

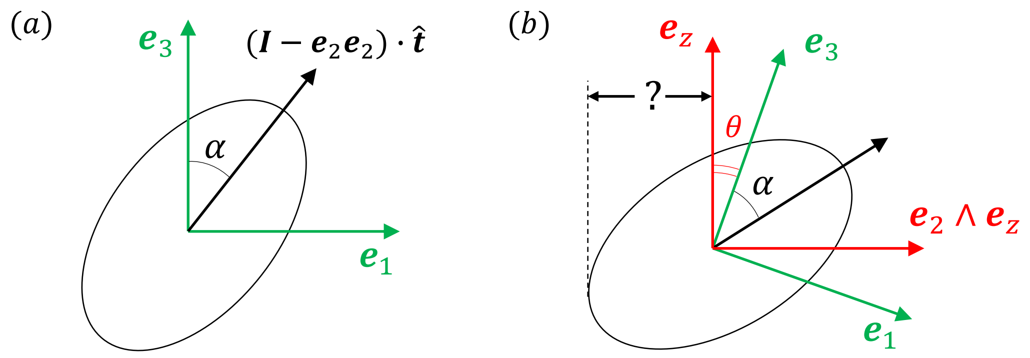

FIG. S2. To a first-order approximation in r_ϵ/r, the cross-sectional shape of the helix in the focal plane is an ellipse. (a) The major axis of the ellipse is parallel to the projection on the focal plane of the tangent vector to the helical centreline. (b) The major axis is inclined at an angle α to the helical axis, $\mathbf{e}_3$, which is in turn inclined at an angle θ to the vertical, $\mathbf{e}_z$. We want to determine the horizontal distance between the extremes of the ellipse and the z-axis.

The major axis of the ellipse is inclined at an angle α to the helical axis, as it is parallel to the vector $\sin\alpha\mathbf{e}_1+\cos\alpha\mathbf{e}_3$ and $\mathbf{e}_3$ is the direction of the helical axis, as seen in Fig. S2a. Using Eqs. (S2) and (S9), we can deduce that the major axis is approximately parallel to $(\boldsymbol{I}-\mathbf{e}_2\mathbf{e}_2)\cdot\hat{\mathbf{t}}(s^*)$. Therefore, the cross-sectional shape of the helical filament is stretched out along the projection of the vector tangent to the centreline onto the focal plane. This is intuitively what we expect.

Next, we compute the distance (in the focal plane) between the filament centreline and the z-axis, denoted $r'_\epsilon(\theta,\phi)$ in the main manuscript. We need to consider the outline of the filament in coordinates along the vectors $\{\mathbf{e}_2\wedge\mathbf{e}_z,\mathbf{e}_z\}$ which span the focal plane. To a first-order approximation this will be an ellipse with minor axis length, $2a$, and major axis length, $2b$, which is inclined at an angle $\theta+\alpha$ to the z-axis. This is because the major axis of the ellipse makes an angle α with the helical axis, which makes an angle θ with the z-axis, as seen in Fig. S2b.

Consider the general problem of finding the horizontal extremes of an ellipse with minor axis length, $2a$, and major axis length, $2b$, inclined at an angle β to the vertical. In Cartesian coordinates (X,Y) relative to the centre of the ellipse, where the Y-axis is the vertical, the ellipse is described parametrically by

$$\begin{pmatrix}X(\zeta)\\Y(\zeta)\end{pmatrix}=a\cos\zeta\begin{pmatrix}\cos\beta\\-\sin\beta\end{pmatrix}+b\sin\zeta\begin{pmatrix}\sin\beta\\\cos\beta\end{pmatrix}. \tag{S18}$$

The horizontal extreme points on the ellipse are the turning points of $X(\zeta)$, so we consider

$$\frac{\mathrm{d}X}{\mathrm{d}\zeta}=-a\sin\zeta\cos\beta+b\cos\zeta\sin\beta=0, \tag{S19}$$

which is solved by ζ^* that satisfies

$$\sin\zeta^*=\frac{b\sin\beta}{\sqrt{a^2\cos^2\beta+b^2\sin^2\beta}},\quad \cos\zeta^*=\frac{a\cos\beta}{\sqrt{a^2\cos^2\beta+b^2\sin^2\beta}}. \tag{S20}$$

Therefore, the horizontal extremes of the ellipse are at a distance

$$X(\zeta^*)=\sqrt{a^2\cos^2\beta+b^2\sin^2\beta} \tag{S21}$$

from the vertical.

Using Eqs. (S17) and (S16) to substitute our own values for a,b and $\beta=\theta+\alpha$, we deduce that

$$r'_\epsilon(\theta,\phi)=r_\epsilon\sqrt{\cos^2\left(\theta+\tan^{-1}(\sin\phi'\tan\psi)\right)+\frac{\sin^2\left(\theta+\tan^{-1}(\sin\phi'\tan\psi)\right)}{\left(\cos\phi'\sin\psi\right)^2}}. \tag{S22}$$

Finally, if we write the denominator of the second term as $\sin^2(\sin^{-1}(\cos\phi'\sin\psi))$, we can simplify the expression for $r'_\epsilon(\theta,\phi)$ to the form found in the main manuscript,

$$r'_\epsilon(\theta,\phi)=r_\epsilon\sqrt{1+\frac{\sin^2\left(\theta+\tan^{-1}(\sin\phi'\tan\psi)\right)}{\tan^2(\sin^{-1}(\cos\phi'\sin\psi))}}, \tag{S23}$$

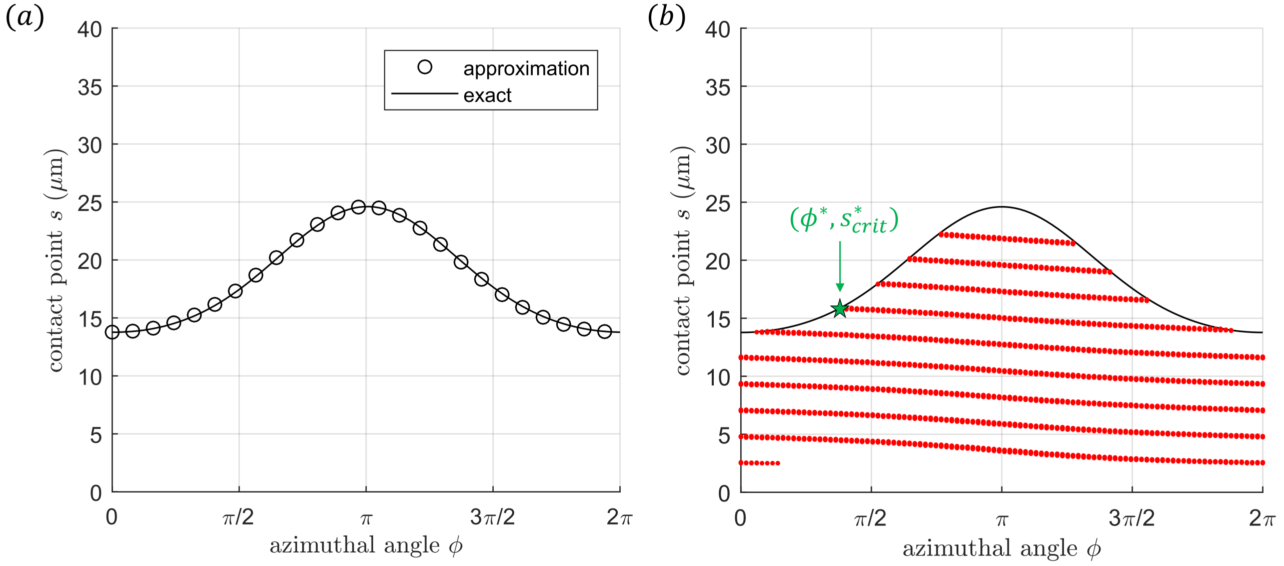

FIG. S3. (a) Representative example of the critical boundary $s_{\rm crit}(\phi)$ for a perfect helix with constant radius. The approximate solution is given by Eqs. (6) and (7) and relies on two small-r_ϵ approximations, while the "exact" solution is based on solving for $r'_\epsilon(\theta,\phi)$ and $\theta_{\rm crit}(\phi)$ numerically. (b) Representative example of the critical boundary $s_{\rm crit}(\phi)$ for a left-handed tapered helix which approaches the same amplitude as the perfect helix in (a). There are additional critical boundaries displayed as red lines, where the first pitch of the helix intersect with the vertical midline. The helical filament used as model for these two diagrams has the same geometric properties as the normal polymorphic form, so the geometric parameters are typical of flagellar filaments in real bacteria.

where we remind that $\phi' = \sin^{-1}(d\sin\phi/2r)$ is an implicit function of ϕ.

III. EFFECT OF TAPERING AND ANCHORING

The theoretical threshold for tangling from Eqs. (6) and (7) is valid for a perfect helix with constant radius r, meaning that the bottom-end $s=0$ is not properly anchored to the point where the helical axis meets the xy-plane – its locus is the hemisphere of radius r above the xy-plane. The global maximum of the critical line $s_{\rm crit}(\phi)$ is at $\phi=\pi$, as seen in Fig. S3a. So, if we wanted to intertwine the helix around the vertical line as much as possible, we could keep the azimuthal angle fixed at $\phi=\pi$ (meaning that the helix is leaning towards the vertical line) and raise the contact point s up to $s_{\rm crit}(\pi)$. As we do this we continuously decrease the phase χ, for which we have the exact solution $\hat{\chi}(s,\phi) = \sin^{-1}(\sigma d\sin\phi/2r) - 2\pi s/p$, while the incline angle $\hat{\theta}(s,\phi)$ gradually decreases. From Eq. (S1), we observe that decreasing the phase χ means that a left-handed helix would be rotating in a left-handed sense and a right-handed helix in a right-handed sense. Therefore, helices of both chiralities would be revolving around the vertical line like a screw, in the sense that is compatible with their geometry.

However, each time the phase χ covers the interval $(0,2\pi)$, the free bottom-end of the perfect helix performs a loop around the vertical line. In fact, any point s_0 will loop around the vertical line once each time we increase χ by a further 2π after the helix was tangent to the line at point $s_0+p/2$, as illustrated in Video S2. This is because the incline angle $\hat{\theta}(\phi,s) < \hat{\theta}(\phi,s_0)$ if $s>s_0$, so the vertical line already lies inside the cylindrical envelope of the helix up to the point s_0. Meanwhile, the point s_0 revolves around this cylindrical envelope once every time the phase $\hat{\chi}(\phi,s)$ covers a period of 2π, which happens each time the contact point s covers an interval of length p.

Suppose we wanted to follow the same strategy (i.e. keep ϕ fixed and increase s) with a tapered helix that is anchored to a fixed point. Then every time χ covers the interval $(0,2\pi)$ we would have additional intersections between the vertical line and the first pitch of the helical filament (or whatever length we assume is sufficient for the filament to approach its full amplitude, say $s_{99\%}$). This is because the end of the filament, $s=0$, is anchored to a fixed location while a point of nearly full amplitude, $s_{99\%}$, loops around the vertical line once, as per our argument for a helix of constant amplitude. The filament is a continuous curve between these two points so it must intersect the vertical line in the process. The intersections can be determined numerically, and are shown as red lines in Fig. S3b. The periodicity of the helix translates into the periodicity of these critical curves, which are separated by vertical gaps of

approximately one pitch. Since they lie on a ϕ-periodic domain, they can be approximated by straight lines of slope $\sigma p/2\pi$ depending on the chirality σ of the helix. For a left-handed helix ($\sigma = -1$) the red lines slope downward, as in Fig. S3b, whereas for the right-handed filament ($\sigma = +1$) in Video S3 the red lines slope upward.

In order to achieve tangling to the highest possible point along the helix, $s^*_{\text{crit}} = s_{\text{crit}}(\phi^*)$, we must navigate the (ϕ, s) space in such a way as to avoid hitting any critical lines. Starting from s close to zero, we may work our way up between the red critical lines until we reach the black critical curve, see Fig. S3b. This trajectory roughly corresponds to keeping the phase χ constant and continuously decreasing the azimuthal angle ϕ. Every time ϕ goes from 2π to 0 the left-handed helix turns around the vertical midline in a clockwise direction, so the two helices are indeed intertwining in a left-handed sense. We calculate numerically the maximum possible height that can be achieved in this way, denoted s^*_{crit}. Note that we allow for the fact that tapering is a modelling concept and therefore its form is not unique. We take the worst possible scenario into account so that we may say with confidence that rigid helical filaments longer than the calculated limit, s^*_{crit}, would not be able to tangle. We assume the lowest red line in Fig. S3b is placed arbitrarily, depending on the form of tapering, but subsequent lines are positioned at fixed vertical gaps of one pitch above it. Then we vary the height of the lowest line in order to calculate the largest possible value for s^*_{crit}, or the worst case scenario.

IV. SOURCES OF EXPERIMENTAL DATA

Table S1 contains a systematic collection of sources from which we gathered the values of our input parameters: the helical geometry (p, r) of polymorphic shapes, the cell body size (l, w), the length, L, and thickness, r_ϵ, of filaments. Our theoretical model for tangling takes these inputs and produces an estimate for the critical number of flagella above which the bacterium may run the risk of tangling. This output must likewise be compared with the number of flagella observed experimentally, from sources also given in Table S1.

We selected strains for which the data could be gathered from as few sources as possible. For each strain we strived to compile a coherent database of compatible sources, preferably coming from the same research group in the same time period. From papers investigating the growth and shape of bacterial flagellar filaments it was possible to gather comprehensive data about the helical geometry of the polymorphic shapes, whereas papers focusing on the speed of locomotion or tumbling frequency often quoted the size of the cell body, and the number and length of flagella. Because the imaging techniques required for measuring the thickness of slender flagellar filaments are distinctly different from the imaging techniques for visualising the entire ensemble of bacterial cell bodies and flagella, it was necessary to use different sources for the filament radius, which were separate from the rest of the database.

For *Salmonella typhimurium* we used Ref. [3] for the pitch and radius of most polymorphic forms because it had the closest links with Ref. [13], from which we took the distributions of filament length and number of flagella. However, this reference did not contain measurements for the semicoiled form, so we tested our theory using measurements from three other experimental groups [4–6]. There was no significant difference between the results obtained with the three different sets of data.

Ref. [7] only provides measurements of the flagellar filament radius for *Salmonella typhimurium* and *Bacillus subtilis*. However, according to Ref. [8], the flagellin in the composition of *Escherichia coli* flagella is very similar in size to that of *Salmonella typhimurium* flagella (497 vs. 489 amino acids) so we assume that the filament radius of the two

**Parameter**	*E. coli* AW405	*E. coli* HCB1	*E. coli* HCB1737	*S. typhimurium* TM2	*B. subtilis* DK2002
pitch, p, and radius, r					
normal form	[1, 2]	[1, 2]	[1, 2]	[3]	[4]
semicoiled form	[2]	[2]	[2]	[4–6]	–
curly I	[2]	[2]	[2]	[3]	[4]
curly II	[2]	[2]	[2]	[3]	–
filament radius, r_ϵ	[7, 8]	[7, 8]	[7, 8]	[7]	[7]
cell length, l	[1]	[9]	[10]	[11]	[10]
cell width, w	[1]	[9]	[10]	[11, 12]	[10]
filament length, L	[1, 2]	[9]	[10]	[13]	[10]
number of flagella, N	[1, 2]	[9]	[10]	[13]	[10]

TABLE S1. Collection of all sources of experimental data used as parameters for our theoretical model, as well as the number of flagella measured in experiments, which was compared with the output of our model.

bacterial species are the same.

Ref. [11] only provides measurements for the cell length of *Salmonella typhimurium*, but in conjunction with Ref. [12] we were able to make a reasonable estimate for the cell width.

V. VIDEO DESCRIPTIONS

Video S1

Visual demonstration that symmetry reduces the problem of two helices of finite thickness tangling around each other to the problem of one helix of finite thickness tangling around a line of zero thickness, which represents the midline between the anchoring points of the original helices. The point of contact between the two helices, or between helix and line, is highlighted by an orange segment.

Video S2

Visual demonstration of the case where a perfect helix tangles around a fixed vertical line. The perfect helix has constant amplitude, r, and its proximal end is not anchored to a fixed point, but free to move on a sphere of radius r centred on the fixed point where the helical axis meets the plane $z = 0$. The motion depicted in this video corresponds to the following trajectory in (ϕ, s) coordinate space: keep the azimuthal angle fixed at $\phi = \pi$ and increase the contact point s monotonically. This trajectory results in the helix being screwed around the vertical line. In the video, we highlight in blue the point $s = p/2$ and in orange the points $s = p, 2p, 3p, 4p$ along the helix. Notice how the point $s = p/2$ revolves around the vertical line once between the time when point $s = p$ and point $s = 2p$ are tangent to the line, then once again by the time point $s = 3p$ becomes tangent to the line, and so on. This illustrates our statement from the main manuscript that each time the contact point covers an interval of length p above some arbitrary point $s_0 + p/2$, the point s_0 loops around the vertical line once.

Video S3

Visual demonstration of the tangling trajectory for two identical, tapered, and anchored helical filaments. On the left we see the symmetric three-dimensional trajectory of the helices, with the point of contact highlighted by an orange segment. On the right we see the trajectory in (s, ϕ) coordinate space, where s is the contact point and ϕ is the azimuthal angle over which the helical axis is inclined. The black curve, $s_{\text{crit}}(\phi)$, represents the constraint imposed by the helical geometry of the helices (see Fig. 3) and was calculated analytically in Eq. (4). The red curves represent the constraints imposed by the anchoring of the helical filaments and were calculated numerically. The blue star represents the current configuration and is shown mounting between the red curves until it reaches the critical boundary, $s_{\text{crit}}(\phi)$. At this point the camera zooms in on the helices and shows that, in addition to the helices touching on the exterior at the point highlighted in orange, they are also touching on the interior at one pitch below it.

[1] N. C. Darnton, L. Turner, S. Rojevsky, and H. C. Berg, "On torque and tumbling in swimming *Escherichia coli*," J. Bacteriol. **189**, 1756–1764 (2007).

[2] L. Turner, W. S. Ryu, and H. C. Berg, "Real-time imaging of fluorescent flagellar filaments." J. Bacteriol. **182**, 2793–801 (2000).

[3] T. Iino, T. Oguchi, and T. Kuroiwa, "Polymorphism in a flagellar-shape mutant of *Salmonella typhimurium*," J. Gen. Microbiol. **81**, 37–45 (1974).

[4] M. Fujii, S. Shibata, and S.-I. Aizawa, "Polar, peritrichous, and lateral flagella belong to three distinguishable flagellar families," J. Mol. Biol. **379**, 273 – 283 (2008).

[5] R. Kamiya and S. Asakura, "Helical transformations of *Salmonella* flagella *in vitro*," J. Mol. Biol **106**, 167–186 (1976).

[6] H. Hotani, "Micro-video study of moving bacterial flagellar filaments: III. Cyclic transformation induced by mechanical force," J. Mol. Biol. **156**, 791–806 (1982).

[7] F. Wang, A.M. Burrage, S. Postel, R. E. Clark, A. Orlova, E. J. Sundberg, D. B. Kearns, and E. H. Egelman, "A structural model of flagellar filament switching across multiple bacterial species," Nature Comm. **8**, 960 (2017).

[8] Shin-Ichi Aizawa, "Topic 4. Flagellin size," in *The Flagellar World*, edited by S.-I. Aizawa (Academic Press, 2014) pp. 54 – 55.

[9] M. Li, *Experimental study of swimming flagellated bacteria and their collective behaviour in concentrated suspensions*, Ph.D. thesis (2010).

[10] L. Turner, L. Ping, M. Neubauer, and H. C. Berg, "Visualizing flagella while tracking bacteria," Biophys. J. **111**, 630–639 (2016).

[11] T. Iino, T. Oguchi, and K. Kutsuka, "Flagellation of *Salmonella typhimurium* treated with nalidixic acid," J. Gen. Microbiol. **133**, 779–782 (1987).

[12] A. Fàbrega and J. Vila, "*Salmonella enterica* serovar typhimurium skills to succeed in the host: virulence and regulation," Clin. Microbiol. Rev. **26**, 308–341 (2013).

[13] T. Iino, "Assembly of *Salmonella* flagellin *in vitro* and *in vivo*," J. Supramol. Struct. **2**, 372–384 (1974).
